# A qualitative analysis of health professionals’ job descriptions for surgical service delivery in Uganda

**DOI:** 10.1186/1478-4491-12-S1-S5

**Published:** 2014-05-12

**Authors:** William Buwembo, Ian G Munabi, Moses Galukande, Olivia Kituuka, Samuel A Luboga

**Affiliations:** 1 Department of Anatomy, School of Biomedical Sciences, Makerere University College of Health Sciences, Uganda; 2 Department of Surgery, School of Medicine, Makerere University College of Health Sciences, Uganda

**Keywords:** job descriptions, surgery, sub-Saharan Africa, Uganda, task shifting, descriptions de tâches, chirurgie, Afrique subsaharienne, Ouganda, délégation des tâches

## Abstract

**Background:**

The ever increasing demand for surgical services in sub-Saharan Africa is creating a need to increase the number of health workers able to provide surgical care. This calls for the optimisation of all available human resources to provide universal access to essential and emergency surgical services. One way of optimising already scarce human resources for health is by clarifying job descriptions to guide the scope of practice, measuring rewards/benefits for the health workers providing surgical care, and informing education and training for health professionals. This study set out to determine the scope of the mandate to perform surgical procedures in current job descriptions of surgical care health professionals in Uganda.

**Methods:**

A document review was conducted of job descriptions for the health professionals responsible for surgical service delivery in the Ugandan Health care system. The job descriptions were extracted and subjected to a qualitative content data analysis approach using a text based RQDA package of the open source R statistical computing software.

**Results:**

It was observed that there was no explicit mention of assignment of delivery of surgical services to a particular cadre. Instead the bulk of direct patient related care, including surgical attention, was assigned to the lower cadres, in particular the medical officer. Senior cadres were assigned to perform predominantly advisory and managerial roles in the health care system. In addition, a no cost opportunity to task shift surgical service delivery to the senior clinical officers was identified.

**Conclusions:**

There is a need to specifically assign the mandate to provide surgical care tasks, according to degree of complexity, to adequately trained cadres of health workers. Health professionals’ current job descriptions are not explicit, and therefore do not adequately support proper training, deployment, defined scope of practice, and remuneration for equitable surgical service delivery in Uganda. Such deliberate assignment of mandates will provide a means of increasing surgical service delivery through further optimisation of the available human resources for health.

## Abstract (fr)

**Résumé:**

**Contexte**

La demande toujours croissante de services chirurgicaux en Afrique subsaharienne crée un besoin d’augmenter le nombre de travailleurs de la santé capables d’offrir des soins chirurgicaux. Cela nécessite l’optimisation des ressources humaines disponibles afin de fournir un accès universel aux services chirurgicaux essentiels et d’urgence. Une façon d’optimiser les ressources humaines en soins de santé déjà rares est de clarifier les descriptions de tâches afin de déterminer les champs de pratique, de mesurer des avantages pour les travailleurs de la santé qui fournissent des soins chirurgicaux et d’uniformiser la formation des professionnels de la santé.

**Objectifs**

Notre étude vise à déterminer la portée du mandat de personnes habilitées à effectuer des procédures chirurgicales dans leur description de tâches existante parmi les professionnels en soins chirurgicaux de l’Ouganda.

**Méthodes**

Une revue des descriptions de tâches a été effectuée pour les professionnels de la santé responsables de la prestation de services chirurgicaux dans le système de santé ougandais. Les descriptions de tâches ont été extraites et soumises à une analyse qualitative de contenu au moyen de l’extension d’analyse textuelle RQDA du logiciel d’analyse statistique en libre accès R.

**Résultats**

Nous avons remarqué qu’il n’existe aucune mention explicite de l’affectation à la prestation de services chirurgicaux à un cadre particulier. La majorité des soins directs aux patients, incluant les soins chirurgicaux, était plutôt assignée au personnel subalterne, en particulier les médecins. Les cadres supérieurs devaient majoritairement effectuer des tâches de conseil ou de gestion dans le système de santé. De plus, nous avons déterminé une façon de déléguer les tâches de chirurgie aux médecins-chefs qui ne coûterait rien.

Il existe un besoin d’assigner les tâches de soins chirurgicaux aux travailleurs de la santé formés en conséquence, en fonction du degré de complexité. Les descriptions de tâches existantes pour les professionnels de la santé ne sont pas explicites, ne soutiennent pas une formation ni un déploiement adéquats et ne définissent pas adéquatement le champ de pratique et la rémunération équitable des services chirurgicaux en Ouganda. Des mandats clairement définis fourniront un moyen d’améliorer la prestation des services chirurgicaux grâce à une optimisation des ressources humaines disponibles en santé.

## Introduction

Surgical conditions in sub-Saharan Africa have been described as the “other” neglected disease in global public health [1]. This is emphasized by the observation that all over the world, but more so in Africa, injuries are identified as a leading cause of loss of DALY among 10-24 year olds [2]. Other forms of documented surgical conditions such as: hernias [3], post abortion care [4], osteomyelitis [5], and the acute abdomen emergency condition that alone is estimated to affect more than 1364/100,000 people per year in Africa, all require surgical attention [6]. The ever increasing demand for surgical services is creating a need to increase the number of people trained in the delivery of surgical care to clear the backlog of cases [3,5-8]. This is the result of the increasing population, increasing numbers of road traffic accidents and other causes of trauma.

One of the solutions suggested, to address this absence of personnel to attend to surgical conditions in sub-Saharan Africa, is through optimisation of available human resources for universal access to essential and emergency surgical services [8]. This optimisation involves the training of lower cadre health workers to perform carefully selected surgical tasks. This has been successfully done in the case of Malawi where non physician clinicians (NPC), have been trained to do caesarean sections at the district hospitals resulting in improved obstetric care [9]. Task optimisation using lower cadre health workers has also been very successful for safe male circumcision in Tanzania [10] and Uganda [11,12]. Several authors have identified the district/general hospital as a key location for attending to these surgical conditions [6,8,13,14]. This health unit is usually staffed by a medical officer who is usually a recent graduate with a bachelor of medicine and surgery degree [8,13]. It has been observed that it is these individuals, as opposed to the trained surgical specialists, who perform the majority of the surgical procedures in sub-Saharan Africa [8]. These are mainly emergency surgical procedures whose number is still far lower than is required to meet the needs of the population served based on the very low proportions of live births born by caesarean section from these hospitals’ catchment areas [13]. This means that even though the district hospitals perform the majority of surgical procedures in the region, there is still a substantial unmet need for surgical services [6,13]. There is no publication linking the quality of surgery to the various providers in these hospitals. In this paper we sought to re-examine the job descriptions of all health carders in relation to their surgical roles as a means of addressing this low output.

Clear job descriptions have been identified as an important factor in both performance and retention of health workers [15,16]. In Malawi, the high patient load coupled with absence or lack of clarity in job descriptions have been identified as one of the reasons the few health workers available commonly overstep their levels of competence [17]. In Mali and Burkina Faso, it has been observed that most of the lower level cadres were not aware of their job descriptions, which are written according to one’s profession and not their posting, resulting in health workers performing tasks for which they are not qualified and in unclear performance appraisals by management [18,19]. These, and other factors including the absence of up-to-date clinical guidelines and long working hours, have been identified as major job related de-motivators and causes of health worker burnout [15,16,20]. Job descriptions define the scope of practice, rewards in the form of re-numeration and recognition, and provide a framework for the appraisal process [15-20]. The clarity of job descriptions will eventually affect the quality of services and the nature of training provided by health professional training institutions [9,15,16]. Given these observations, the project team identified exploration of the available job descriptions as an additional opportunity for optimising the available human resources for surgical care in Uganda. We are not aware of any prior study attempting to elucidate the implications of health professionals’ job descriptions for addressing surgical service delivery in Uganda.

By 2012 there were 97 specialist surgeons and another 107 trained Ugandan general surgeons and 124 gynaecologists and obstetricians for a population of 35 million people [21]. Table 1 summarises the current staffing norms and other descriptive background information for the various health facilities and carders mentioned in this study [22]. It is important to note that health centre IV units are the lowest health facilities in the Ugandan health systems structure, ideally managed by a medical officer, with infrastructure for emergency surgery, for a population of 100,000 people. In 2009 it was observed that 56% of these health centre IVs had a functional theatre, and only 16% a functional surgical ward [23]. There was an improvement from 22% in 2009 [23] to the current 36% of the health centre IV facilities offering emergency caesarean sections in the 2012/2013 financial year [22]. This information on performance of these health centre IV units is important given that the primary policy objective of establishing them was to make emergency surgical services like the emergency caesarean section more accessible to the general population. One other challenge identified in the 2009 report, in relation to human resources for health, was the high turnover of staff. This was highest for medical officers at 65% attrition rates in private not-for-profit hospitals, meaning that these hospitals were operating with the least experienced human resources [23]. In the annual health sector performance report of 2012, the most frequently given reason for departure was a search for better terms of employment [24]. This paper explores the assignment of responsibility for surgical service delivery in the job descriptions of surgical care health professionals in Uganda.

**Table 1:** Descriptive information of health workers in Uganda.

Item	Hospital					Populat-ion per health worker	Post secondary training (years)
	Salary scale (annual start salary in UGX)*	HCIV	General	Regional	National		

Number	-	166	47	13	2	-	-

Vacancies (%)	-	40	43	28	10.5	-	-

Population covered	-	100,000	500,000	2,000,000	35,000,000	-	-

Clinical officer	U5c 3,250,000	2	4	20	50	5583	3

Medical officer	U5a-3 6,500,000	1	3	10	50	7272	5

Surgeons	U3 7,600,000	-	1	5	30	171,568	8

Obstetrician	U3 7,600,000	-	1	5	40	282,258	8

Number of health workers and vacancies based on Annual Health Sector Performance Report (2012). Exchange rate US$1 = 2500 UGX. Services provided: HCIV: preventive, promotive outpatient curative, maternity, inpatient health services, emergency surgery and blood transfusion and laboratory services; General: in addition to services offered at HCIV, other general services will be provided. It will also provide in service training, consultation, and research to community based health care programmes; Regional: in addition to services offered at the general hospital, specialist services will be offered, such as psychiatry, ear, nose and throat, ophthalmology, dentistry, intensive care, radiology, pathology, higher level surgical and medical services; National: these provide comprehensive specialist services and are involved in teaching and research.

## Methods

The analysis for this paper was based on a document review of the job descriptions of all health professionals responsible for general surgical care in the Ugandan health care system. Other methods like focus group discussions, surveys and in depth interviews generate data based on respondent’s interpretation of the job descriptions. Such interpretations should follow an in depth analysis of the job descriptions as described in this paper. Included in the study analysis were the job descriptions of clinical officers, medical officers, surgeons, and obstetricians. In the Uganda health care system, the lowest cadre are the clinical officers who are usually non degree holding health workers, sometimes referred to as physician assistants in other settings, with four years of hands-on post-secondary education. In Uganda, medical officers are bachelor degree holders in medicine and surgery while the surgeons and obstetricians have an additional three years of training to attain a master’s degree in surgery and obstetrics, respectively, after the bachelor’s degree (see tables 1 and 2). The master’s degree holders supervise the medical and clinical officers according to their terms of employment as consultants. Medical officers may, with experience, supervise the clinical officers.

**Table 2:** Advertised job descriptions. Adapted from the District Service Commission of Uganda’s health vacancies notice [30].

Cadre	Duties defined	Other Requirements	Missing
**Medical Officer**	Participates in diagnosis, treatment and management of patients in the Assessment centre, Casualty, Clinics and Wards Evaluates patients and refers them for specialized health care Participates in the delivery of quality health care to patients Adheres to the relevant Code of conduct and Ethics Participates in outreach and community health programmes Participates in research activities and health data collection Compiles and submits periodic reports Participates in Continuing Professional Development Programme Performs any other duties as may be assigned from time to time	Qualification: Bachelor of Medicine and Surgery (MBChB) or its equivalent, from a recognized University / institution	Surgical role is not mentioned

**Medical Officer Special Grade – Surgery (Surgeons)**	Responsible for ensuring efficient management of service in general surgery at the National Referral Hospital and the catchment area covered by the Hospital Supervises and mentors Senior, House Officers, Interns and other Health Professionals under him/ her Plans and coordinates the training programmes of all the Health Professionals under him / her Plans and coordinates continuous professional Development (CPD) in his/ her speciality, for Health professionals at the National Referral Hospital and the catchment area of the Hospital Provides technical and professional advice in general surgery to Government and the National Referral Hospital Performs any other duties as may be assigned from time to time	Qualification: MBChB or its equivalent from a recognized University or institution And a Master of Medicine degree in Surgery or its equivalent from a recognised University or Institution Applicant must have demonstrable qualities of leadership and integrity	Patient care related surgical role is not mentioned

**Medical Officer Special Grade – Obstetrics & Gynaecology (Obstetricians)**	Responsible for ensuring efficient management of service in Obstetrics & Gynaecology at the National Referral Hospital and the catchment area covered by the Hospital Supervises and mentors Senior, House Officers, Interns and other Health Professionals under him/ her Plans and coordinates the training programmes of all the Health Professionals under him / her Plans and coordinates continuous professional Development (CPD) in his/ her speciality, for Health professionals at the National Referral Hospital and the catchment area of the Hospital Provides technical and professional advice in Obstetrics & Gynaecology to Government and the National Referral Hospital Performs any other duties as may be assigned from time to time	Qualification; MBChB or its equivalent from a recognized University or institution And a Master of Medicine degree in Obstetrics & Gynaecology or its equivalent from a recognised University or Institution Applicant must have demonstrable qualities of leadership and integrity	Patient care related surgical role is not mentioned

**Clinical officer**	i. Diagnosing, treating and managing patients; ii. Conducting health education to patients; iii. Participating in research activities; iv. Participating in Continuous Professional Development activities; and v. Preparing and submitting reports.	Must have a Diploma in Clinical Medicine and Community Health or its equivalent from recognized Institution Must be registered and licensed with the Allied Health Professionals Council.	Surgical role is not mentioned

A search was made for job descriptions of health professionals practicing in Uganda. The search involved visiting the institutional bodies mandated with employment of health workers which are the Uganda Health Service Commission and the Uganda Ministries of Public Service and Local Government [25,26]. The Uganda Health Service Commission is a statutory body mandated by law to advertise, interview, and later appoint health professionals in Uganda to various government health facilities. According to the decentralisation policy of Uganda, the Ministries of Public Service and Local Government coordinate the recruitment activities of local governments to address the health needs of the rural population [26]. Only documents that already exist in the public domain, for the years 2007-2012, and those used to attract potential applicants, were included in the study.

Excluded from analysis were job descriptions of health professionals employed by the non-governmental organisations (e.g. projects, faith based health care providing institutions, and private health care facilities) as these serve a specific clientele as opposed to the general population. The other reason for excluding these institutions’ job descriptions is the current practice by government of seconding health professionals to these units, in line with the private-public partnership agreements, to support increased health service delivery in the country [27]. This implies that for a health worker to be employed in these institutions they must also satisfy the public sector job descriptions.

The job descriptions were extracted for four cadres of health workers: clinical officer, medical officer, surgeon, and obstetrician. They then underwent qualitative content data analysis, aided by a text based open source RQDA package in the R statistical analysis software [28,29]. The job descriptions were typed verbatim into a word processor and saved as text files, compared with the original text to ensure no errors, prior to exporting to the RQDA package. The descriptions were coded and later themes were identified. Memos and annotations were used to capture additional observations for each item with respect to the researchers’ experiences as practicing health professionals. Reading and coding of the job descriptions was repeated several times until no new codes or themes were identified. The analysis was conducted by two of the researchers independently, with analysis for levels of agreement. A third researcher was called in to resolve any conflicts arising during analysis.

Ethical approval for this study was obtained from the Makerere University School of Medicine, Research and Ethics Committee at Kampala, Uganda.

## Results

The document search identified job descriptions for the targeted health professionals archived in the form of one newspaper advertisement [30] and a job description document for local governments from the Ministry of Public Service and Local Government [31]. Table 1 is a verbatim extract from the newspaper job descriptions advertisement [30] from the Uganda Health Services Commission for the four targeted study groups of health workers (clinical officers, medical officers, surgeons, and obstetricians). It is of interest to note that the newspaper advert had less detail compared to the document from the Ministry of Public Service. This may have been due to the cost implications of using advertising space in the print media. The public service document had details on: who the person reports to, responsibilities, required competencies, key outputs, key functions, and salary scale. Analysis of the identified job descriptions generated three distinct themes. The theme of direct patient care captured codes relating to activities/tasks that put the individual in direct contact with the patient. This was observed with the medical officer and the clinical officer job descriptions. Included in direct patient care, with a total of 13 codings, were: participation in community programs (n=2), doing emergency work (n=1), referral of patients (n=1), research and data collection (n=2), and patient activities (n=7) described as: “*Diagnose*, *treat and manage patients in the Health Unit.”*

A second theme was for items that indirectly impact patient care (with a total of 39 codings). The indirectness arose from the observations that these items result in change of action or behaviour of other professionals/systems that then impact patient care. This was observed with the more qualified/experienced health professionals: surgeons, obstetricians, and senior medical officers. Under indirect patient care, the following was detected: advice to the hospital or government (n=2), training other professionals, mainly through organising continuing medical education sessions (n=4), qualifications (n=12), and management, with 19 codings. In the district job descriptions, years of experience on the job are a major requirement for promotion to the next level. The management related tasks and expectations increase with seniority demonstrated by the senior medical officer’s tasks/outputs; only 1/15 are directly patient care related. The importance of the management role is further emphasised in the list of competencies required for the senior health professionals, which are strongly biased towards management.

The competencies for the senior medical officer are “*Planning*, *Organizing and coordinating; Project Management; Information management; Accountability; Concern for quality and standards; Ethics & integrity; Teamwork; Communication; Report writing; and Leadership.”* Note the absence of one’s clinical/surgical skills as a required competence.

The final theme looked at job-related items (with a total of 20 codings). Included in this theme were: the salary scale (n=8), persons the individual is responsible for (n=8), and being assigned other duties (n=4). Regarding salary scale, both the medical officer and the senior clinical officer are placed at the same salary scale, which is still lower than the scale for senior medical officers, surgeons and obstetricians. An additional observation from the job descriptions was that the senior clinical officer is responsible for coordinating a team of clinical officers while the medical officer did not have any cadre assigned to them to supervise. The only mention of a surgical-related working space in all the job descriptions included in the study was in relation to casualty for the medical officer in a referral hospital (see table 1). For the surgeons and the obstetricians, emphasis is placed on responsibility of “*ensuring efficient management of service*” in either surgery or obstetrics as opposed to the actual performing surgical related tasks. This further emphasises the management role of these cadres.

## Discussion

We set out to explore the assignment of responsibility for surgical service delivery in the job descriptions of surgical care health professionals in Uganda and found that surgery, or operations, or surgical theatres, where surgery takes place, are not explicitly mentioned in these job descriptions. The closest reference to this is “casualty” for the medical officer working in the emergency unit of the national referral hospital which has an operating theatre by the same name. One could argue that the use of the term “management” in the task of “*Diagnose*, *treat and manage patients in the Health Unit*,” includes surgery. Alternatively, surgery as a form of specialised care would qualify for referral according to the task “*Evaluates patients and refers them for specialized health care”* (Table 1: medical officer). This omission regarding the surgical roles of those health professionals could lead to health workers excusing themselves from performing surgical tasks that are often tedious and time-consuming. On the other hand, the same medical officer could end up performing an operation that is beyond his/her level of competence. This lack of clarity in the job descriptions has been identified as a cause of stress and loss of job satisfaction in various studies [32,33]. In the Pacific and Asian region, this type of workload-related stress was associated with a high turnover of health workers [33]. Complaints about the workload are already being observed in Uganda and have been associated with high turnover of health workers in health facilities, as was mentioned earlier with regards to attrition of medical officers from private not-for-profit hospitals [22-24,34,35]. While most of these studies seem to suggest that the cause of dissatisfaction has to do with the health workers’ remuneration, infrastructure, supplies, location of the facility, and benefits, we posit that the lack of clarity in the job descriptions could be another factor leading to job dissatisfaction among health workers.

We observed that the job descriptions clearly assign most of the patient care work to the lower and less experienced health professionals, as shown by the more frequent coding (n=20) of these patient care related tasks/activities for the clinical and medical officers. In the case of surgery, this falls on the shoulders of the medical officer. The more senior health workers, that is the senior medical officer, surgeons and obstetricians, move into administrative ranks where emphasis is placed on giving advice, management, and training, as evidenced by the higher frequency (n=39) of coding for administrative related tasks/activities. This could lead to a further emphasis on the already existing hierarchy in the health care system and also partly explain the better retention of health professionals at the senior ranks, as seen in other low and middle income countries of the Asian and Pacific region [33]. Senior physicians may also be reducing their surgical work, given the number of administrative tasks/activities assigned to them, combined with local politics and low numbers of human resources for health [34]. The absence of the health professionals’ clinical/surgical skills as one of the desired competences for senior medical officers, the immediate supervisors of medical and clinical officers, the heavy workload/low pay, coupled with long working hours, could motivate junior medical officers to quickly work towards promotion or departure to better settings [22-24,33,34,36]. This had been noted with the case of the private not-for-profit hospitals in Uganda with the 2012 annual health sector report stating the most frequently given reason for departure being the search for better terms of service for junior medical officers [24]. In their current state, the job descriptions may be creating several training related challenges. Some of these challenges include: ensuring lower cadre health workers have the correct skill sets for surgery, and adequately tooling the senior health professionals for their management and mentorship roles [37]. Currently, the surgical training for medical officers, at the undergraduate level, focuses more on exposure to key surgical skills [38]. This, however, is changing with the increasing recognition of the medical officer’s role in providing surgical services, as evidenced by the changes in the way the internship program is run in Uganda, to ensure attainment of key surgical skills for medical officers to run the theatre at the health centre IV level [22-24,39,40].

Finally, the observation that the medical officer is at the same salary scale as a senior clinical officer (who is not supposed to perform any surgery), may be a further disincentive for the medical officer to perform surgical procedures. From our experience and anecdotal evidence, the medical officer is often responsible for the nursing/surgical care teams, and sometimes the clinical officers, by virtue of the nature of their medical training. From our experience, it is also common to find that the medical officer also doubles as the accounting officer for the unit, and as such, has a supervisory role over the clinical officer. This same medical officer may spend the night working on surgical emergency procedures (e.g. caesareans). This creates a disparity with respect to payment for performance and has been a cause of contention in the Ugandan public health care system for a long time [34,36,41]. For these medical officers, the time spent preparing and attending these administrative meetings has been identified as one of the reasons for lost productivity with respect to patient care at the health centre IV level [42,43]. There are even calls for separation of these management and patient roles for the medical officer at this level, to enhance patient care productivity as is seen with medical officers in general hospitals [43]. For the senior health professionals, the added opportunity to spend time in private practice is a further disincentive for them to perform their supervisory and training roles [33]. There are currently efforts to change this by rewarding the medical officer for working in remote and hard to reach parts of the county [36]. There is no additional reward for the number of or time spent doing surgical procedures.

In their current format, the job descriptions for surgical health workers create both challenges and opportunities for improved surgical service delivery. The observation that the bulk of surgical care is left to the medical officer running the health centre IV unit creates an opportunity to optimise available human resources for universal access to essential and emergency surgery by transferring assignment of surgical responsibility from the specialist physician to non-specialist physician [22-24,37]. This, with proper training from the health professional training institutions, provision of up-to-date guidelines, hands-on surgical mentorship by specialist surgeons on a longitudinal basis, a clear supervision and performance appraisal process, and accompanying remuneration for extra hours, should result in increased access to quality surgical service delivery [15-17,27]. In the district health care system, the practice of rewarding years of service with promotion, in the form of vertical loading, may need revision to add an emphasis on recognizing/rewarding ones’ performance of surgical tasks and mentorship [15,16]. This could be done by separating the management roles of these senior health workers from their clinical service provision roles. Given the very low numbers of trained specialist physician surgeons (204) [21], and the fact that most health units are at 50% or less of the establishment for medical officers are filled [22-24], there is an additional opportunity to explore training the senior clinical officer to take on the added role of providing key surgical services at no additional cost to the health system in the form of salary payments. Given the bigger numbers of this cadre at the lower units (see table 1), this could lead to increased access to surgical services, as has been the case in other countries like Malawi that have applied surgical task shifting. Prior to full scale roll out of such an initiative there is a need to address the ethical, quality, and legal concerns of such an intervention in Uganda [17]. Finally, while reviewing the job descriptions, it could be advantageous to create a single harmonized document for the entire health care delivery system, for all settings. This would allow for the matching of types of cases seen at the different levels in the system with health worker cadres of the desired level of surgical training and skills, and create clear career paths in the system [38].

Limitations of the study include the use of a limited number of information sources, the advertised job descriptions and the job description document from the Ministry of Public Service and Cabinet Affairs. Future studies could explore the views and interpretations by the various health professionals, as was the case in Malawi [17]. This additional exploration, coupled with actual workplace time motion studies, may strengthen the observations made in this paper.

## Conclusions

There is a need to specifically assign the mandate of surgical care in the current job descriptions of health professionals, to support proper training, deployment, defined scope of practice and remuneration for equitable surgical service delivery in Uganda. Such deliberate assignment of mandate will provide for a means of increasing surgical service delivery through further optimisation of the available human resources for health. The detailed description of tasks to be performed, and personal/educational specifications for each cadre of health workers, will help ensure that surgical work is performed by those to whom the responsibility has been assigned. This could be a major source of motivation for the performance of surgical services, even in remote and underserved parts of Uganda.

## Recommendation

An effort to specifically define the surgical responsibilities of each cadre health worker should be undertaken, to provide guidelines for the training, supervision, appraisal, promotion, remuneration, and recognition of health workers involved in surgery in Uganda.

## List of abbreviations

DALY: disability-adjusted life year; DFATD: Foreign Affairs, Trade and Development Canada; GHRI: Global Health Research Initiative; IDRC: International Development Research Centre; NPC: non physician clinicians; RQDA: R package for computer assisted qualitative data analysis

## Competing interests

The authors declare no competing interests.

## Authors’ contributions

IGM, BW, LAS and MG participated in the conceptualization of the paper. IGM, LAS, BW, OK and MG drafted and provided scientific reviews of the manuscript. All authors participated in the review and development of the final submitted manuscript.

## Declarations

The publication costs associated with this article are funded by Foreign Affairs, Trade and Development Canada and the International Development Research Centre through the Global Health Research Initiative.

This article has been published as part of *Human Resources for Health* Volume 12 Supplement 1, 2014: Addressing the human resources for health crisis through task-shifting and retention: results from the Africa Health Systems Initiative's research component. The full contents of the supplement are available online at http://www.human-resources-health.com/supplements/12/S1
